# Monoclonal Antibodies against Accumulation-Associated Protein Affect EPS Biosynthesis and Enhance Bacterial Accumulation of *Staphylococcus epidermidis*

**DOI:** 10.1371/journal.pone.0020918

**Published:** 2011-06-07

**Authors:** Jian Hu, Tao Xu, Tao Zhu, Qiang Lou, Xueqin Wang, Yang Wu, Renzheng Huang, Jingran Liu, Huayong Liu, Fangyou Yu, Baixing Ding, Yalin Huang, Wenyan Tong, Di Qu

**Affiliations:** 1 Key Laboratory of Medical Molecular Virology of Ministries of Education and Health, Institute of Medical Microbiology and Institutes of Biomedical Sciences, Fudan University, Shanghai, China; 2 Department of Intensive Care Unit, the First Affiliated Hospital of Wenzhou Medical College, Wenzhou, China; 3 Institute of Stem Cell Research and Regenerative Medicine and Institutes of Biomedical Sciences, Fudan University, Shanghai, China; Baylor College of Medicine, United States of America

## Abstract

Because there is no effective antibiotic to eradicate *Staphylococcus epidermidis* biofilm infections that lead to the failure of medical device implantations, the development of anti-biofilm vaccines is necessary. Biofilm formation by *S. epidermidis* requires accumulation-associated protein (Aap) that contains sequence repeats known as G5 domains, which are responsible for the Zn^2+^-dependent dimerization of Aap to mediate intercellular adhesion. Antibodies against Aap have been reported to inhibit biofilm accumulation. In the present study, three monoclonal antibodies (MAbs) against the Aap C-terminal single B-repeat construct followed by the 79-aa half repeat (AapBrpt1.5) were generated. MAb_18B6_ inhibited biofilm formation by *S. epidermidis* RP62A to 60% of the maximum, while MAb_25C11_ and MAb_20B9_ enhanced biofilm accumulation. All three MAbs aggregated the planktonic bacteria to form visible cell clusters. Epitope mapping revealed that the epitope of MAb_18B6_, which recognizes an identical area within AapBrpt constructs from *S. epidermidis* RP62A, was not shared by MAb_25C11_ and MAb_20B9_. Furthermore, all three MAbs were found to affect both Aap expression and extracellular polymeric substance (EPS, including extracellular DNA and PIA) biosynthesis in *S. epidermidis* and enhance the cell accumulation. These findings contribute to a better understanding of staphylococcal biofilm formation and will help to develop epitope-peptide vaccines against staphylococcal infections.

## Introduction

*Staphylococcus epidermidis*, an opportunistic pathogen, has become one of the most prevalent causes of nosocomial infections, especially in patients with prosthetic medical devices [1], [2]. *S. epidermidis* colonization of these devices is complicated by the formation of biofilms, which render it increasingly resistant to multiple antibiotics and host defenses [3], [4]. Replacement of the indwelling medical devices after *S. epidermidis* biofilm infection is generally necessary, and the development of biofilm-preventing vaccines is imperative.

Biofilms are bacterial communities that adhere to biological or abiotic substrata and are stabilized by extracellular polymeric substances (EPSs), typically composed of polysaccharides and extracellular DNA [2], [5], [6], [7], [8]. The formation of staphylococcal biofilms involves two phases: primary adhesion followed by biofilm accumulation [4], [9], [10], [11]. Once attached to the substrata, the bacteria will proliferate, secrete and be enmeshed within EPS, and then accumulate as multilayered cell clusters. Polysaccharide intercellular adhesin (PIA), which is synthesized by proteins encoded in the *ica* operon [12], [13], [14], [15], [16], and extracellular DNA (eDNA) released from dead bacteria [6], [7], [8] have been considered essential in the process of staphylococcal biofilm accumulation. However, *ica*-negative but biofilm-positive staphylococci have recently been described, with biofilms do not contain PIA but rely solely on protein-protein interactions [17], [18]. Accumulation-associated protein (Aap) is considered one of the most important proteins that involved in *S. epidermidis* biofilm formation. Implicated in both polysaccharide-based [19] and protein-based [17], [20] biofilms, Aap can directly mediate intercellular adhesion. According to an amino acid sequence analysis, Aap contains an A region and a B-repeat region. The A region, containing an N-terminal A-repeat domain with 11 degenerate 16-aa repeats and a putative globular domain (“α/β”), has been found to mediate the adhesion of *S. epidermidis* to human corneocytes [21]. The B-repeat region (AapBrpt), composed of a variable number (5 to 17) [20] of nearly identical 128-aa repeat constructs terminating in a conserved “half repeat” motif, promotes intercellular adhesion [17], [18] through Zn^2+^-dependent dimerization [22].

Antiserum against Aap showed inhibition of both proteinaceous [17], [20] and polysaccharide-based [19] biofilm formation by *S. epidermidis*. Biofilm-inhibiting monoclonal antibodies against Aap have also been established [23]. Aap could be considered a vaccine candidate to prevent biofilm infections. In the present study, mouse monoclonal antibodies (MAbs) raised against the Aap C-terminal single B-repeat construct followed by the 79-aa half repeat (AapBrpt1.5), which has been proven to be the basic functional unit required for Aap to mediate bacterial accumulation [22], were prepared to locate the epitopes that induce the production of anti-biofilm antibodies for the further development of epitope-peptide vaccines. However, only MAb_18B6_ inhibited biofilm formation by *S. epidermidis* RP62A to 60% of the maximum, whereas MAb_25C11_ and MAb_20B9_ enhanced biofilm accumulation. Epitope mapping revealed that MAb_18B6_ recognized an identical area within all AapBrpt constructs, which was not shared by MAb_25C11_ and MAb_20B9_. The effects of the MAbs on Aap expression and EPS biosynthesis in *S. epidermidis* were further studied to investigate the enhanced biofilm formation and bacterial accumulation. Our study provides new insights into the mechanisms of staphylococcal biofilm formation and may help in developing anti-staphylococcal biofilm vaccines.

## Results

### General characteristics of the MAbs against AapBrpt1.5

**Figure 1:**
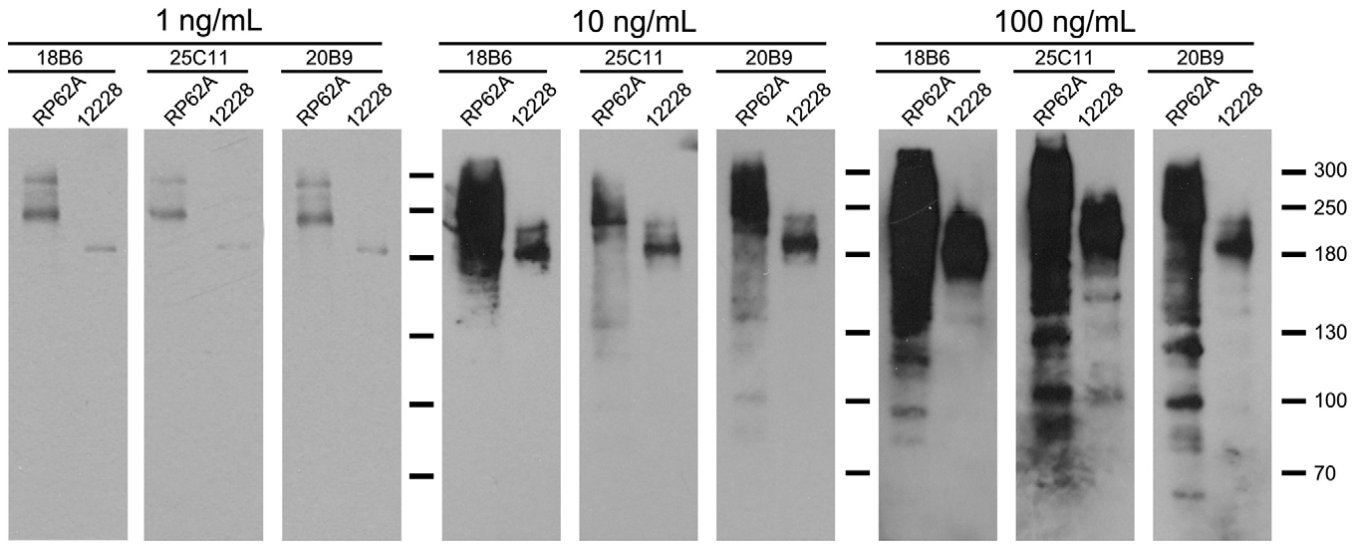
Antigenic specificity of the MAbs. The antigenic specificities of the MAbs at 1 ng/mL and at higher concentrations (10 ng/mL, 100 ng/mL) against proteins in supernatants of lysostaphin-treated *S. epidermidis* RP62A (“RP62A”) and ATCC 12228 (“12228”) were analyzed using Western blot. The multiple proteins (between 250 kDa–300 kDa) in *S. epidermidis* RP62A, or the single protein (180 kDa) in *S. epidermidis* ATCC 12228, probed by 1 ng/mL MAbs, corresponded to full-length or proteolytically processed Aap, based on an analysis of these bands using a 4700 MALDI-TOF/TOF proteomics analyzer (Applied Biosystems, http://www.appliedbiosystems.com).

To locate the epitopes of the anti-biofilm antibodies, three mouse monoclonal antibodies against AapBrpt1.5 from *S. epidermidis* ATCC 12228 were prepared and termed MAb_18B6_, MAb_25C11_, and MAb_20B9_. All three MAbs, purified using protein G-Sepharose from mouse ascites, were identified as IgG. The immunoreactivity of the MAbs was detected using enzyme-linked immunosorbent assay (ELISA) and immunoprecipitation. The MAbs bound to recombinant AapBrpt1.5 with a high affinity (ELISA titers ≥1∶1,280,000 per 0.4 mg/mL antibody), and the MAbs interacted with AapBrpt1.5 under both non-denaturing and denaturing conditions. Moreover, at a low concentration (1 ng/mL), the MAbs bound specifically to Aap in *S. epidermidis*, whereas they interacted with multiple proteins at higher concentrations (≥100 ng/mL), as detected by Western blot (Figure 1).

### Anti-AapBrpt1.5 MAbs recognize different epitopes

**Figure 2:**
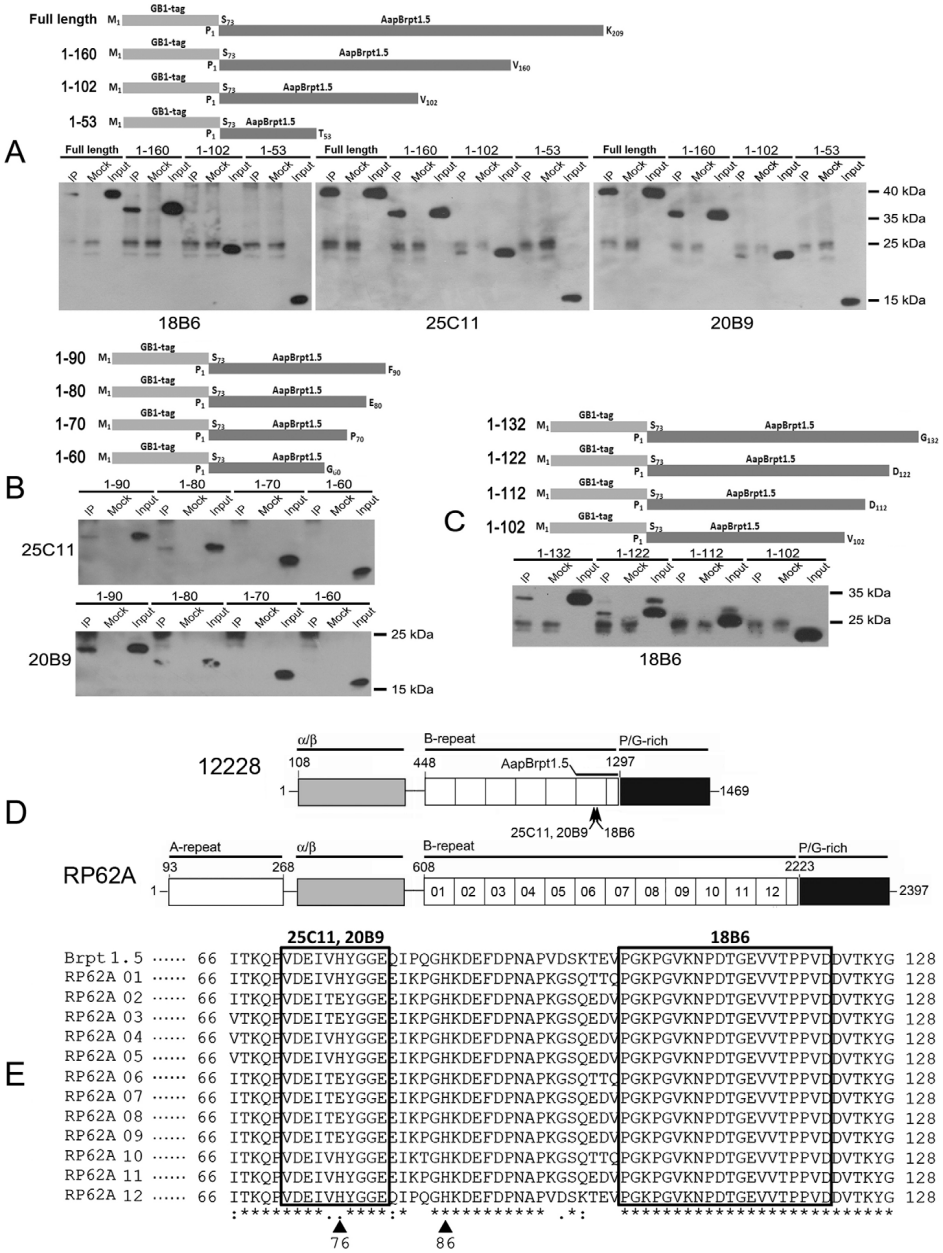
Epitope mapping of anti-AapBrpt1.5 MAbs. AapBrpt1.5 N-terminally fused with a GB1-tagged six-histidine (GB1-His) tag was truncated into a series of fragments as shown in the schematic diagrams, and the binding ability between the truncated fragments and MAbs was analyzed using immunoprecipitation. (A) Preliminary epitope mapping. (B) Precise recognition site mapping of MAb_25C11_ and MAb_20B9_. (C) Precise recognition site mapping of MAb_18B6_. (D) Domain structures of Aap from *S. epidermidis* RP62A (“RP62A”) and ATCC 12228 (“12228”). The A-repeat region, the putative globular domain (α/β), the B-repeat region containing 6 or 12 tandem Brpt constructs, the collagen-like proline/glycine-rich region, the domain boundary of AapBrpt1.5, and the MAb epitopes are illustrated. (E) Amino acid sequence alignment of AapBrpt constructs. The AapBrpt construct in AapBrpt1.5 (GenBank NP_763730) and twelve distinct AapBrpt constructs from *S. epidermidis* RP62A (RP62A 01-12, GenBank YP_189945) were aligned using the ClustalW2 program (http://www.ebi.ac.uk/Tools/clustalw2). The identified epitopes of the MAbs are shown in boxes, and the identical residues are marked with asterisks. The conserved substitutions are represented by “:”, and semi-conserved substitutions are represented by “.”. Two conserved His residues in AapBrpt constructs are marked with triangles.

To locate the epitopes of the MAbs, AapBrpt1.5 (N-terminally fused to a GB1-His-tag [24], [25], [26]) was truncated into the following fragments: TF_1–160_, TF_1–102_, and TF_1–53_ (Figure 2A). The interactions between truncated fragments and the MAbs were studied using immunoprecipitation. MAb_18B6_ interacted with the truncated fragment TF_1–160_ but not the others, and MAb_25C11_ and MAb_20B9_ interacted with both TF_1–160_ and TF_1–102_ (Figure 2A), indicating that the recognition site for MAb_18B6_ is located between aa 103–160 and the sites for MAb_25C11_ and MAb_20B9_ are located between aa 54–102. In addition, truncated fragments of AapBrpt1.5, TF_1–132_, TF_1–122_, TF_1–112_, TF_1–90_, TF_1–80_, TF_1–70_, and TF_1–60_, were prepared for more precise mapping (Figure 2B, C). The precise epitopes of MAb_25C11_ and MAb_20B9_ were located between aa 71–80 (Figure 2B), which are in a non-identical area within AapBrpt constructs from *S. epidermidis* RP62A (Figure 2D, E). Regarding MAb_18B6_, its recognition site was located within aa 103–122 (Figure 2C), which is identical to the homologous position in all 12 AapBrpt constructs from *S. epidermidis* RP62A (Figure 2D, E).

### Anti-AapBrpt1.5 MAbs affect the biofilm formation by *S. epidermidis*

**Figure 3:**
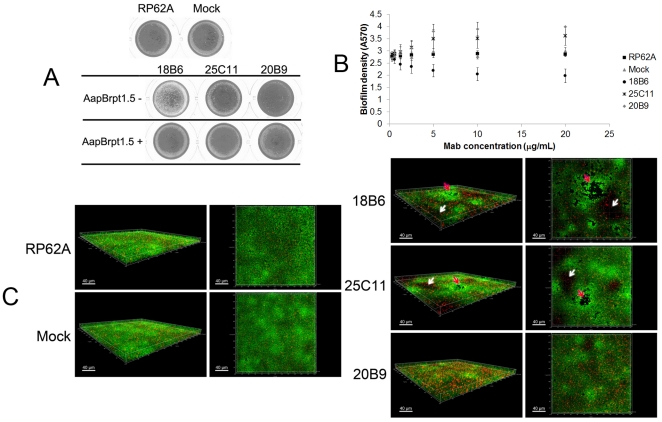
Anti-AapBrpt1.5 MAbs affected biofilm formation by *S. epidermidis*. (A) Macroscopic profiles of the biofilms cultured in polystyrene plates. The biofilms were cultured in TSB medium containing each MAb (10 µg/mL, 0.07 µM) alone or both MAbs (10 µg/mL, 0.07 µM) and AapBrpt1.5 (untagged, 3.2 µg/mL, 0.14 µM). The images represent one of three independent experiments. (B) Anti-AapBrpt1.5 MAbs affected biofilm formation by *S. epidermidis* RP62A in a dose-dependent manner. The biofilm formation was measured using crystal violet staining, and the results are depicted as means ± SD of three independent experiments. (C) Three-dimensional structures of 14-h-old biofilms. Biofilm formation by *S. epidermidis* RP62A in the presence of each MAb (10 µg/mL) was visualized using Live/Dead viability staining (SYTO9/PI) and observed under a confocal laser scanning microscopy (CLSM). Green fluorescent cells are viable, whereas red fluorescent cells are dead. The images, representing one of three independent experiments, were three-dimensionally reconstructed using Imaris software (Bitplane, http://www.bitplane.com) based on CLSM data at approximately 0.5 µm increments. “RP62A”: untreated, “Mock”: normal mouse IgG-treated; white arrows and red arrows indicate thin areas and crater-like micropores, respectively.

By assaying biofilm formation in polystyrene plates [17], [19], all three MAbs were found to affect the biofilm formation by *S. epidermidis* RP62A: MAb_18B6_ inhibited biofilm formation to 60% of the maximum, while MAb_25C11_ and MAb_20B9_ enhanced the biofilm accumulation (Figure 3A, B). Twice the molar amount of AapBrpt1.5 added to the MAbs completely abolished the ability of the antibodies to affect biofilm formation (Figure 3A). Moreover, normal biofilm of *S. epidermidis* (with a depth of 4.5 µM) had a smooth surface and contained proportionally more viable cells and less dead bacteria (Figure 3C). However, the surface of the biofilm formed in the presence of MAb_18B6_ and MAb_25C11_ (especially MAb_18B6_) was rough, and the biofilm (with a depth of 6.5 µM) contained many crater-like micropores and thin areas (Figure 3C). The biofilm formed in the presence of MAb_20B9_ was much thicker (10.5 µM) than the untreated one (Figure 3C). All biofilms formed in the presence of the MAbs had a higher proportion of dead cells, as demonstrated using the fluorescence quantity ratios (Table 1).

**Table 1:** Fluorescence quantities of the Live/Dead stained biofilms.^a^

MAbs	STTO9^b^	PI^c^	Total^d^	PI/Total^e^
18B6	745.6	159.0	906.9	0.175
25C11	1010.1	247.2	1258.8	0.196
20B9	1018.3	512.8	1531.1	0.335
Mock	1228.1	205.3	1435.0	0.143
-	1430.5	191.5	1623.4	0.118

**a**. The biofilm of *S. epidermidis* RP62A formed in the presence of each MAb (10 µg/mL) was visualized using Live/Dead viability staining (SYTO9/PI). After obtaining the 3-D structure of the biofilms under a CLSM (Figure 3), the Z-stack composite confocal photomicrographs were further generated, and the stacks of viable cells, dead cells, and both cells (viable & dead) were generated separately. The fluorescence quantities of SYTO9-stained viable cells and PI-stained dead cells were determined using ImageJ program (http://rsbweb.nih.gov/ij).

**b**. “SYTO9” stands for the fluorescence quantities of the viable cell stacks.

**c**. “PI” stands for the fluorescence quantities of the dead cell stacks.

**d**. “Total” represents the fluorescence quantities of both cell stacks.

**e**. “PI/Total” represents the proportion of the dead cell in the biofilms.

**Figure 4:**
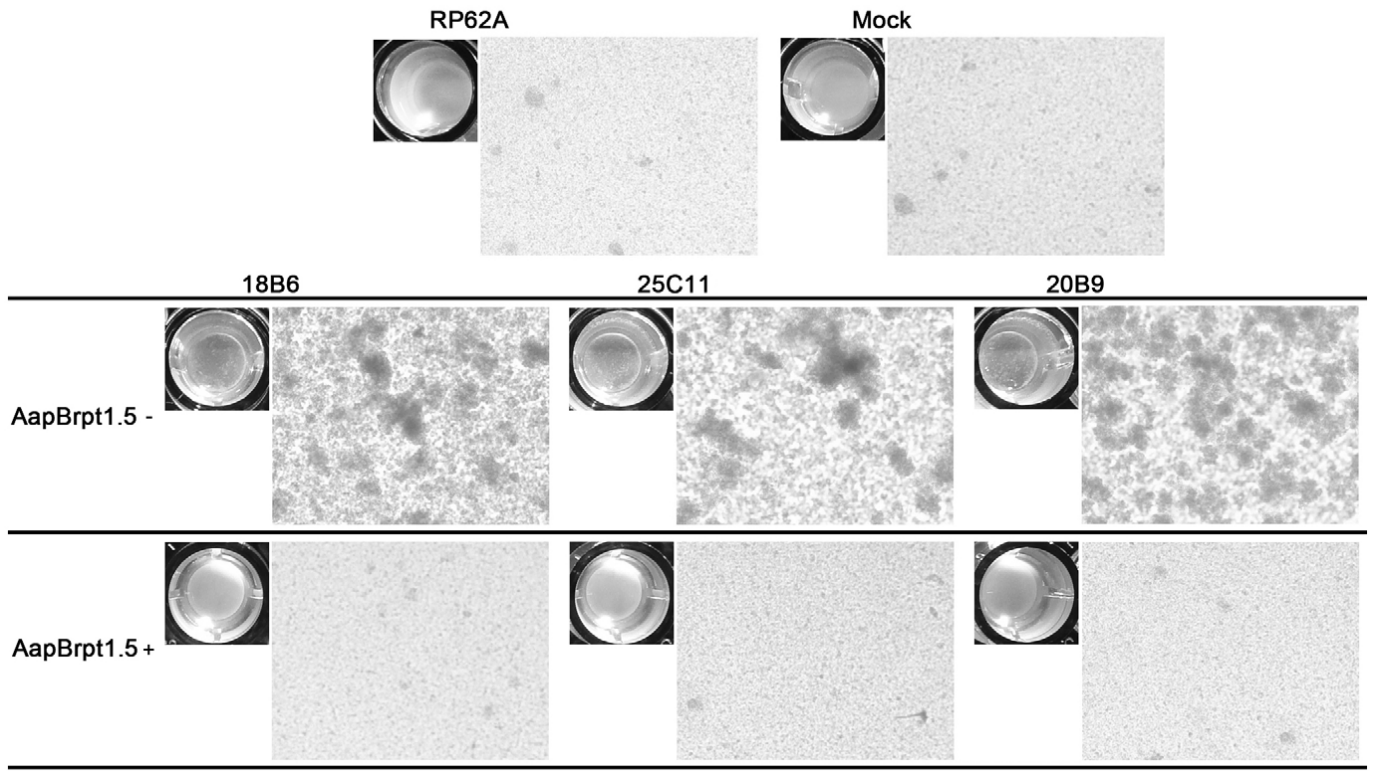
Cell aggregation mediated by the MAbs. *S. epidermidis* RP62A was statically cultured in TSB medium containing each MAb (10 µg/mL, 0.07 µM) alone or both MAb (10 µg/mL, 0.07 µM) and AapBrpt1.5 (untagged, 3.2 µg/mL, 0.14 µM). The photomicrographs were obtained by a Nikon TE2000-U inverted microscopy using a 40x objective lens (Nikon, http://www.nikoninstruments.com/). “RP62A”: untreated; “Mock”: normal mouse IgG-treated.

In addition to biofilm formation, planktonic bacteria of *S. epidermidis* RP62A co-cultivated with the MAbs formed macroscopically and microscopically visible cell clusters, and the aggregation of the cells was initiated at 9 h post-incubation. Twice the molar amount of AapBrpt1.5 added to the MAbs abolished the ability of the antibodies to aggregate the bacteria (Figure 4). To analyze whether the formation of the cell clusters was due to immune agglutination, the concentration of the MAbs contained in the bacterial culture was evaluated by SDS-PAGE (silver staining). The MAbs in bacterial culture was found to be degraded with time, and it could not be detected after 10 h post-incubation (data not shown). It indicated that the cell aggregation was not mediated by immune agglutination because the formation of the clusters was initiated at 9 h post-incubation while the MAbs was almost undetected. Moreover, the aggregated planktonic cells were completely disaggregated upon treatment with proteinase K, whereas DNase I and sodium-meta-periodate (used to rule out the involvement of eDNA or polysaccharide, respectively) had less effect on disintegrating the clusters, suggesting that formation of the cell clusters might be related to the up-regulated expression of intercellular adhesion-associated proteins, probably including Aap.

### Anti-AapBrpt1.5 MAbs affect Aap expression in *S. epidermidis*

**Figure 5:**
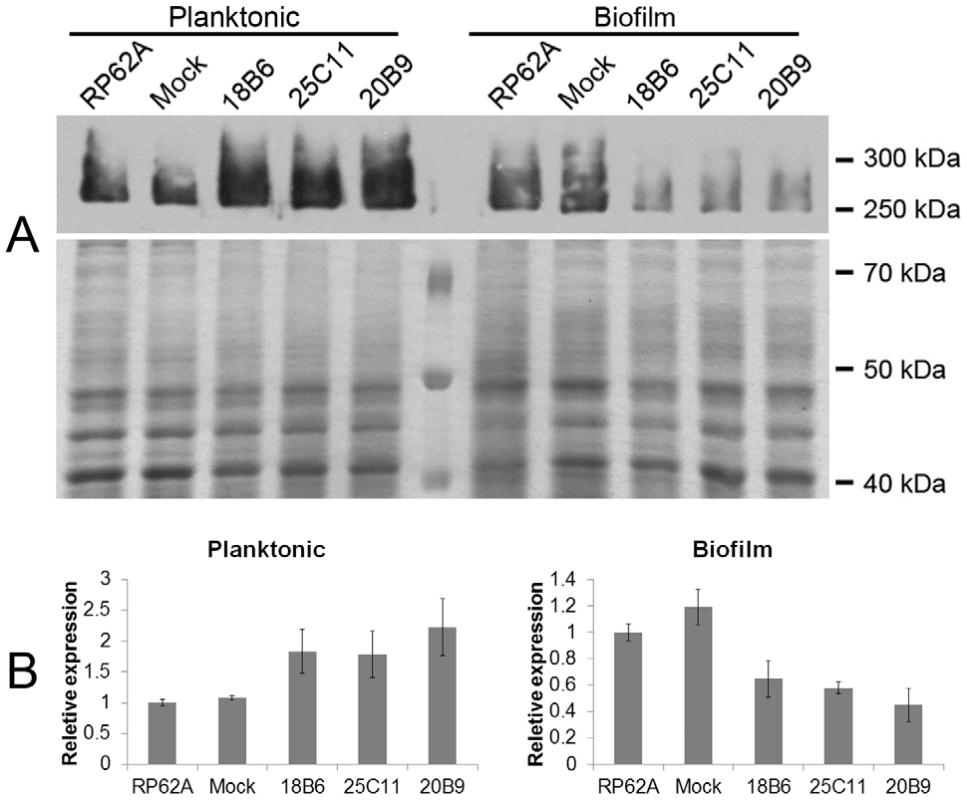
Aap expression in *S. epidermidis*. (A) Protein expression level. Aap expression in *S. epidermidis* RP62A co-cultured with each MAb (10 µg/mL) was detected using Western blot with MAb_25C11_ (1 ng/mL). After separation of the proteins using 7% SDS-PAGE, the gel pieces carrying high molecular-weight proteins (>130 kDa) were excised for Western blot assay, and the remaining gel was stained using Coomassie brilliant blue as the endogenous control. (B) Relative transcriptional level. The transcriptional level of *aap* was detected by Q-RT-PCR using RNA sample extracted from *S. epidermidis* RP62A co-cultured with each MAb (10 µg/mL). The housekeeping gene *gyrB* was used as an endogenous control, and all samples were analyzed in triplicate and normalized against *gyrB* transcription (means ± SD). “RP62A”: untreated, “Mock”: normal mouse IgG-treated.

**Figure 6:**
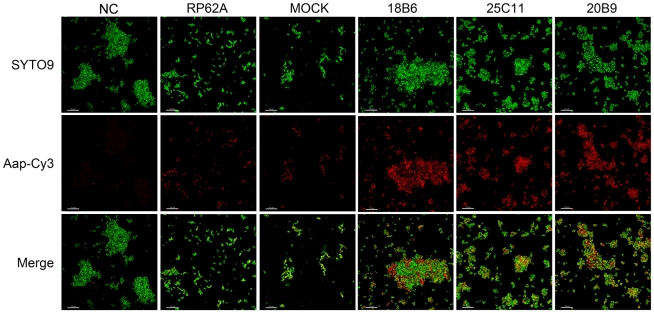
Aap expression in planktonic *S. epidermidis*. Aap expressed in planktonic *S. epidermidis* RP62A (cultured for 14 h) was probed with MAb_25C11_ (10 ng/mL) and Cy3-conjugated secondary antibody (1∶100 diluted, red). The bacteria were stained with SYTO9 (1 µM, green) and observed under a Leica TCS SP5 CLSM. “NC”: negative control (the cell clusters formed in the presence of MAb_25C11_ were probed with Cy3-conjugated secondary antibody alone to establish that the clusters no longer contained initially added MAb (10 µg/mL), that could cause false-positive immunofluorescence, after 14 h culture), “RP62A”: untreated; “Mock”: normal mouse IgG-treated.

**Figure 7:**
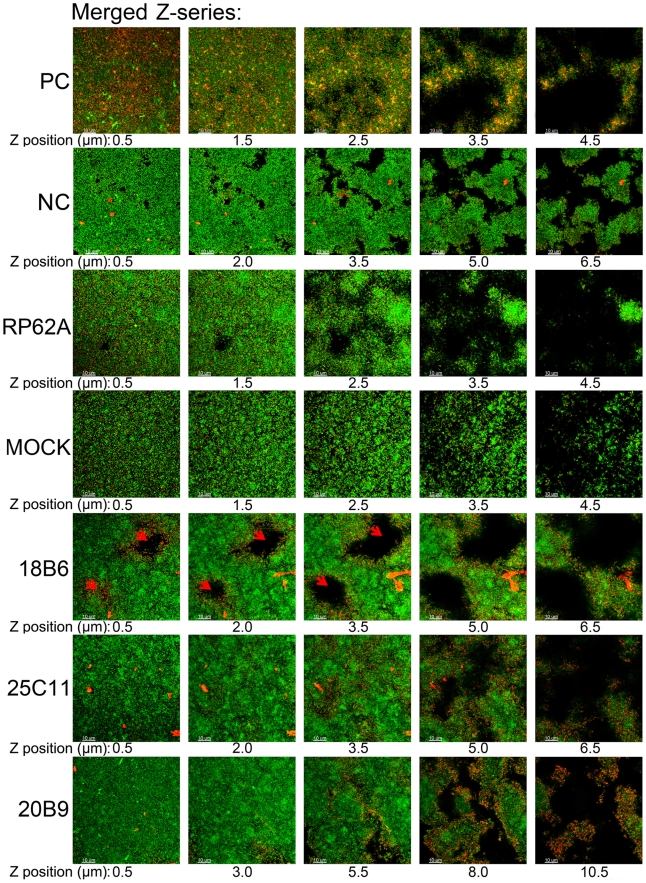
Aap expression in biofilms of *S. epidermidis*. Aap in the biofilms of *S. epidermidis* RP62A was probed with MAb_25C11_ (10 ng/mL) and Cy3-conjugated secondary antibody (1∶100 diluted, red fluorescence), and the bacteria were further stained with SYTO9 (1 µM, green fluorescence). Aap expression was observed under a Leica TCS SP5 CLSM. Confocal microscopy Z-series of the biofilms were acquired in 0.5-µm increments. “PC”: positive control (antigens contained in the biofilm were probed using mouse anti-*S. epidermidis* serum (1∶400 diluted) and Cy3-conjugated secondary antibody, showing that antibodies could diffuse to the inner side of the biofilm), “NC”: negative control (the biofilm formed in the presence of MAb_25C11_ was probed with Cy3-conjugated secondary antibody alone to establish that the MAb-treated biofilms no longer contained initially added MAb (10 µg/mL), that could cause false-positive immunofluorescence, after 14 h culture), “RP62A”: untreated, “Mock”: normal mouse IgG-treated, the red arrow indicates the crater-like micropores.

The Aap expression in *S. epidermidis* RP62A co-cultured with anti-AapBrpt1.5 MAbs was studied using Western blot and immunofluorescence. In the absence of the MAbs, planktonic and biofilm *S. epidermidis* expressed similar amounts of Aap (Figure 5), whereas, notably, planktonic cells co-cultured with the MAbs showed up-regulated Aap expression (Figure 5, 6 and Table 2). Bacteria in superficial layers of the MAb-treated biofilms and the cells at the boundaries of crater-like micropores in MAb_18B6_-treated biofilm also expressed more Aap (Figure 7 and Table 3), whereas the Aap expression of bacteria in profound layers of the biofilms co-incubated with the MAbs was obviously down-regulated (Figure 5, 7 and Table 3).

**Table 2:** Fluorescence quantities of the immuno-probed planktonic cells.^a^

MAbs	SYTO9^b^	Cy3^c^	Cy3/SYTO9^d^
18B6	234.9	168.1	0.716
25C11	267.2	135.0	0.505
20B9	255.6	184.8	0.723
Mock	71.8	19.5	0.272
-	133.3	32.8	0.246

**a**. The planktonic cells of *S. epidermidis* RP62A and Aap were stained by SYTO9 and Cy3, respectively. After obtaining the fluorescence photomicrographs under a CLSM (Figure 6), the fluorescence quantities of SYTO9-stained cells and Cy3-stained Aap were determined using ImageJ program.

**b**. “SYTO9” stands for the fluorescence quantities of SYTO9-stained cells.

**c**. “Cy3” stands for the fluorescence quantities of Cy3-stained Aap.

**d**. “Cy3/SYTO9” represents the proportion of Aap in the cells.

**Table 3:** Fluorescence quantities of the immuno-probed biofilms.^a^

MAbs	Z-position (**µ**m)	SYTO9^b^	Cy3^c^	Cy3/SYTO9^d^
18B6^e^	0.5	1382.8	99.0	0.072
	2.0	1668.6	127.7	0.077
	3.5	1797.4	365.1	0.203
	5.0	1666.0	797.7	0.479
	6.5	984.7	630.4	0.640
25C11	0.5	1043.1	43.1	0.041
	2.0	1250.7	107.5	0.086
	3.5	1253.3	356.2	0.284
	5.0	739.8	402.5	0.544
	6.5	218.4	163.3	0.748
20B9	0.5	764.2	23.0	0.030
	3.0	907.8	44.3	0.049
	5.5	735.8	165.8	0.225
	8.0	417.7	295.3	0.707
	10.5	151.4	194.1	1.282
Mock	0.5	712.1	211.6	0.297
	1.5	779.8	225.8	0.290
	2.5	803.9	245.7	0.306
	3.5	559.5	165.7	0.296
	4.5	201.1	51.8	0.258
-	0.5	1040.6	378.5	0.364
	1.5	1133.4	415.2	0.366
	2.5	950.4	345.5	0.364
	3.5	399.6	129.1	0.323
	4.5	128.2	37.6	0.293

a. The biofilm of *S. epidermidis* RP62A and Aap were stained by SYTO9 and Cy3, respectively. After obtaining the fluorescence photomicrographs under a CLSM (Figure 7), the fluorescence quantities of SYTO9-stained cells, Cy3-stained Aap were determined using ImageJ program.

b. “SYTO9” stands for the fluorescence quantities of SYTO9-stained cells.

c. “Cy3” stands for the fluorescence quantities of Cy3-stained Aap.

d. “Cy3/SYTO9” represents the proportion of Aap in the biofilms.

e. Fluorescence quantities of SYTO9 and Cy3 in biofilm co-cultured with MAb_18B6_ were obtained from other images (not shown) which captured at the area away from the boundaries of the crater-like micropores where Cy3-stained Aap was up-regulated in all Z-positions.

### Anti-AapBrpt1.5 MAbs affect the eDNA release and PIA biosynthesis in *S. epidermidis*

**Figure 8:**
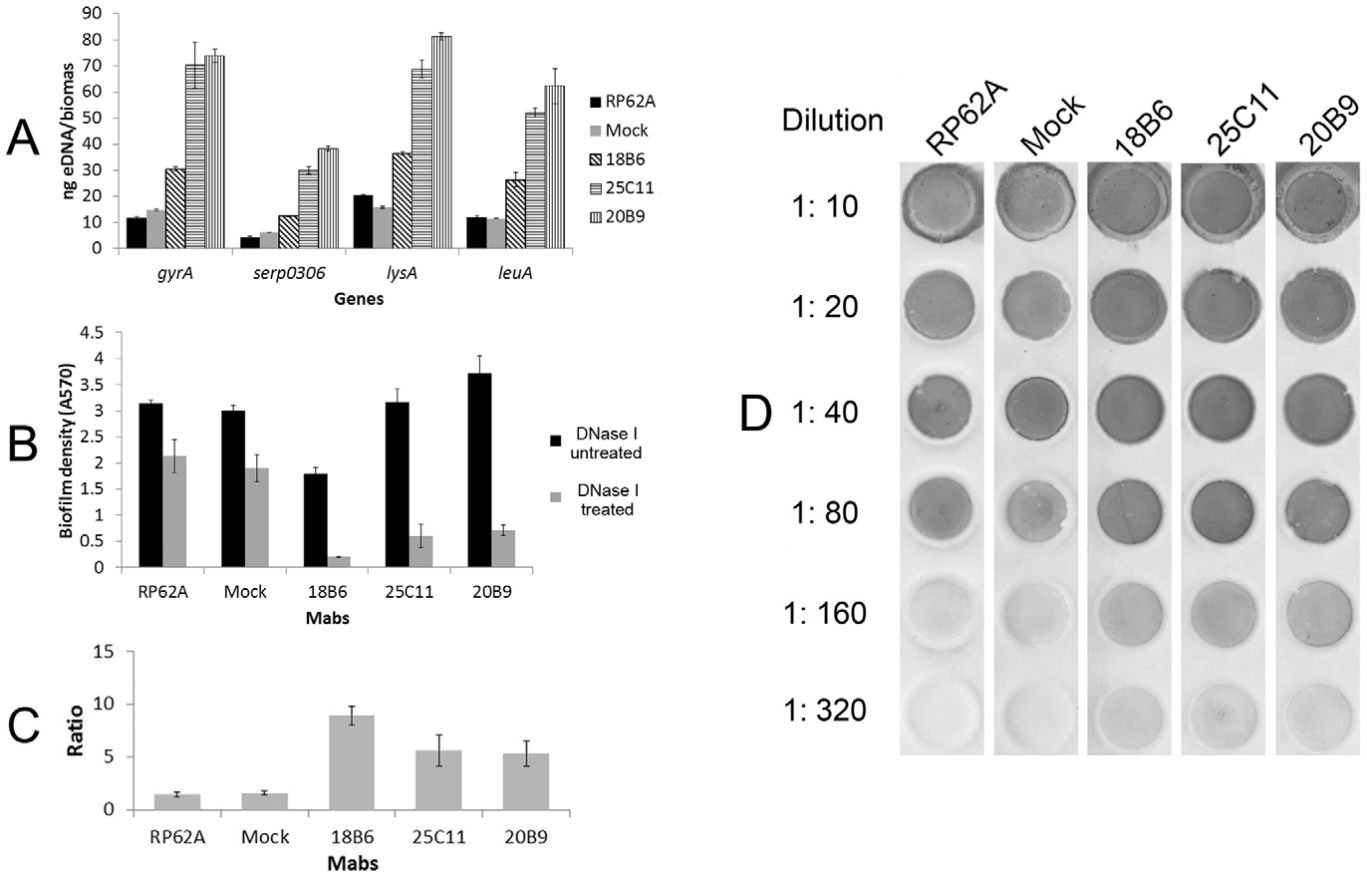
EPS biosynthesis in biofilm of *S. epidermidis*. (A) Extracellular DNA quantification. Extracellular DNA was isolated from the biofilm matrices of *S. epidermidis* RP62A, and Q-PCRs of four chromosomal loci (*gyrA* (gyrase A), *serp0306* (ferrichrome transport ATP-binding protein A), *lysA* (diaminopimelate decarboxylase A), and *leuA* (2-isopropylmalate synthase)) were performed for eDNA quantification in each biofilm. The biomass that represented the biofilm density was quantified at A_600_, and the eDNA measurement was normalized to biofilm biomass as described previously [27]. Data are depicted as averages of three Q-PCR detections with the standard deviation, and the results represent one of three independent experiments. (B, C) Biofilm stability against DNase treatment. When exposed to DNase I (0.14 U/µL), the biofilm was more severely disintegrated in the presence of each MAb compared with that formed in the absence of the MAbs. The data are means ± SD of three independent experiments. The biofilm density ratios of the untreated biofilms and biofilms treated with DNase I were plotted (C). (D) PIA synthesis in biofilm of *S. epidermidis*. PIA synthesis in biofilm of *S. epidermidis* RP62A was detected using the WGA-HRP dot blot assay. Serial dilutions of the PIA extractions from biofilm bacteria were spotted onto nitrocellulose transfer membranes, and the HRP activity was visualized using chromogenic detection. The data represent one of three independent experiments. “RP62A”: untreated; “Mock”: normal mouse IgG-treated.

Because eDNA and PIA are essential to staphylococcal biofilm formation in addition to Aap [6], [7], [12], [13], [14], [15], [16], the biosynthesis of these two EPSs in biofilms co-cultured with the MAbs was analyzed. Quantitative PCRs (Q-PCRs) of four chromosomal loci (*gyrA*, *lueA*, *lysA*, and *serp0306*) were performed to detect the eDNA quantity in the biofilms [27], [28], [29]. The eDNA release from biofilms formed in the presence of the MAbs (especially for MAb_25C11_ and MAb_20B9_) was obviously up-regulated (Figure 8A). Furthermore, when treated with DNase I, the biofilm formed in the presence of the MAbs was more severely disintegrated than that formed in the absence of the antibodies (Figure 8B, C). The up-regulated eDNA release was consistent with the higher proportion of dead cells in biofilms co-cultured with the MAbs (Table 1). However, no significant Triton X-100-induced autolysis of *S. epidermidis* RP62A treated with the MAbs was observed (data not shown) compared with the untreated one. In addition, PIA synthesis in biofilms co-cultured with the MAbs was also up-regulated (Figure 8D), as detected using a wheat germ agglutinin (WGA)-horseradish peroxidase (HRP) dot blot assay [30], [31], [32].

## Discussion

Antibodies against Aap have been shown to inhibit biofilm formation [17], [19], [20], [23], indicating that Aap may serve as a vaccine candidate to prevent *S. epidermidis* biofilm infections [33], [34]. However, full-length Aap is not a safe vaccine for systemic immunization because such bacterial antigens contain many antigenic determinants and may induce hypersensitivity reactions [35], [36]. A peptide that induces anti-biofilm humoral immunity would be an optimal vaccine. Previous studies have shown that AapBrpt1.5 is the basic functional unit of Aap required to mediate the bacterial accumulation [22], suggesting that AapBrpt1.5 should harbor the epitopes that would guide the development of biofilm-preventing epitope-based peptide vaccines. Monoclonal antibodies against AapBrpt1.5 were prepared in the present study to identify these epitopes.

### The anti-AapBrpt1.5 MAbs have different effects on biofilm formation

Because of the difficulties in cloning *aapBrpt1.5* from genome of *S. epidermidis* RP62A, AapBrpt1.5 from *S. epidermidis* ATCC 12228, which shares 92.3% identical residues with that from *S. epidermidis* RP62A, was cloned and expressed, and three MAbs against AapBrpt1.5 were generated. MAb_18B6_ inhibited biofilm formation by *S. epidermidis* RP62A to 60% of the maximum, while MAb_25C11_ and MAb_20B9_ enhanced the biofilm accumulation (Figure 3). The effects of the MAbs on biofilm formation by other laboratory and clinical strains of *S. epidermidis* were further deciphered. MAB_18B6_ exhibited obvious biofilm-inhibiting effect on RP62A, C328, and C847, whereas it showed a mild effect on 1457 and C408. MAb_25C11_ and MAb_20B9_ enhanced the biofilm formation by RP62A, 1457, and C408, whereas they weakly inhibited the biofilm formation by C328 and C847 (Table 4). Additionally, anti-AapBrpt1.5 MAbs showed little effect on biofilm formation or planktonic aggregation of biofilm-negative strains (Table 4).

**Table 4:** Effects of anti-AapBrpt1.5 MAbs on different *S. epidermidis* strains.

	Strain general features			Relative biofilm formation (%)^a^			Planktonic aggregation^b^		
Strains	*ica*	Biofilm formation^c^	Planktonic aggregation^d^	MAb_18B6_	MAb_25C11_	MAb_20B9_	MAb_18B6_	MAb_25C11_	MAb_20B9_
RP62A	+	++	−	66.8	120.0	132.8	+++	+++	+++
1457	+	++	−	84.0	151.3	143.8	+++	+++	+++
C408^e^	+	++	−	91.5	107.9	122.5	+	+	+
C328^e^	+	++	−	70.0	83.9	97.7	+++	+++	+++
C847^e^	+	+	+	53.8	64.8	67.3	++	++	++
C698^e^	+	+/−	−	−	−	−	−	−	−
12228	−	−	−	−	−	−	−	−	−

**a.** “Relative biofilm formation” of the strains cultured in the presence of each MAb (10 µg/mL) in polystyrene plates was calculated by dividing the mean density of MAb-treated biofilms by that of untreated biofilms. “-” means no biofilm formation was observed.

**b.** Planktonic aggregation of the strains statically cultured in the presence of each MAb (10 µg/mL) was described using “+++”, “++”, “+”, or “−”, which means that very many, many, a few, or no bacterial clusters were observed, respectively.

**c.** Biofilm formation by the strains without treatment was described using “++”, “+”, “+/−”, or “−”, which means that strong, weak, slight, or no biofilm formation was observed in polystyrene plates, respectively.

**d.** Cell aggregation of the strains without treatment was described using “+” or “−”, which means that a few or no bacterial clusters were observed, respectively.

**e.** The clinical strains of *S. epidermidis* were obtained from Zhongshan Hospital and Ruijin Hospital, Shanghai, China.

### The anti-AapBrpt1.5 MAbs recognize different epitopes

**Figure 9:**
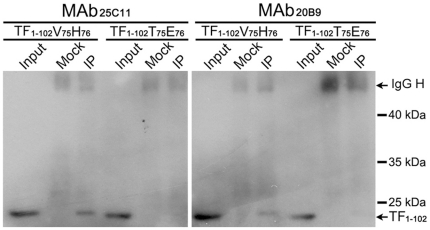
The substitution of V_75_H_76_ by T_75_E_76_ in TF_1–102_ reduced the interaction between the fragment and MAb_25C11_ and MAb_20B9_. The AapBrpt1.5 fragment, TF_1**–**102_, with the substitution of V_75_H_76_ by T_75_E_76_ was established using site-directed mutagenesis. The interactions between the mutated fragment and MAb_25C11_ and MAb_20B9_ were detected using immunoprecipitation. “TF_1–102_V_75_H_76_”: wild type, “TF_1–102_T_75_E_76_”: mutant, “IgG H”: IgG heave chain.

The presumed critical sites of Aap to mediate intercellular adhesion are thought to be two histidine residues (His_76_ and His_86_) in AapBrpt constructs (marked with triangles in Figure 2E) [22], and the binding sites of our MAbs were determined to be nearby these His residues (Figure 2D, E). Because there is no structural report on the AapBrpt construct, it is difficult to determine how these MAbs affect Aap dimerization by binding to their epitopes. Nevertheless, the biofilm-inhibiting activity of the MAbs should be related to their binding sites. MAb_18B6_ showed efficient biofilm-inhibiting activity, and its epitope was found in all AapBrpt constructs from *S. epidermidis* RP62A. MAb_25C11_ and MAb_20B9_ exhibited less biofilm inhibition than MAb_18B6_, and their epitopes were found in only six constructs of AapBrpt while the other constructs possessed two amino acid substitutions (V_75_H_76_ to T_75_E_76_) (Figure 2E). In addition, the substitution of V_75_H_76_ by T_75_E_76_ in an truncated AapBrpt1.5 fragment (TF_1–102_) obviously reduced the interaction between the fragment and MAb_25C11_ and MAb_20B9_ (especially for MAb_25C11_, Figure 9), providing further support that MAb_25C11_ and MAb_20B9_ does not bind to Brpt constructs with the V_75_H_76_ substituted and they cannot block Aap dimerization completely.

### The anti-AapBrpt1.5 MAbs affect Aap expression, eDNA release, and PIA synthesis and enhance the bacterial accumulation

To investigate the reasons for the biofilm-enhancing activities of MAb_25C11_ and MAb_20B9_, Aap expression in *S. epidermidis* RP62A co-cultured with the MAbs were further studied. Detecting by Western blot and immunofluorescence, Aap expression in planktonic cells and superficial biofilm bacteria treated with the MAbs was found to be up-regulated, while the expression in bacteria in profound layers of the biofilm was down-regulated (Figure 5, 6, 7 and Table 2, 3). Although the bacteria in superficial layers of the biofilm co-incubated with the MAbs expressed more Aap (to 2∼4 fold, Table 3), the obviously reduced Aap expression in profound layers (to 0.1∼0.2 fold, Table 3) was consistent with the down-regulated biofilm Aap expression detected by Western blot (Figure 5). The up-regulated Aap expression in planktonic and superficial biofilm cells could mediate increased intercellular adhesion, which aggregated the planktonic bacteria to form cell clusters and further assisted more cells to accumulate on the biofilm surface. Therefore, the biofilm cultured with the MAbs was thicker (6.5–10.5 µM) than that without treatment (4.5 µM). Moreover, the thickened boundaries of the crater-like micropores (Figure 3C) in MAb_18B6_-treated biofilm may also relate to the local up-regulated Aap expression (Figure 7). In addition to Aap expression, eDNA release (Figure 8A, B, C) and PIA synthesis (Figure 8D) were also up-regulated in bacteria co-cultured with the MAbs, resulting in further enhanced bacterial accumulation. Therefore, the up-regulated Aap expression, eDNA release and PIA synthesis enhanced the biofilm formation.

### Two contradictory actions of the MAbs on biofilm formation

Up to now, we have revealed two contradictory actions of the MAbs on biofilm formation. For one, the MAbs block Aap dimerization by binding to AapBrpt constructs and thereby inhibit bacterial accumulation and biofilm formation, and the effect is decreased with time due to degradation of the MAbs. For the other, the MAbs up-regulate Aap expression and EPS biosynthesis of the bacteria, which result in enhanced bacterial accumulation and biofilm formation, and the effect should be, contrarily, evoked and increased with time.

**Figure 10:**
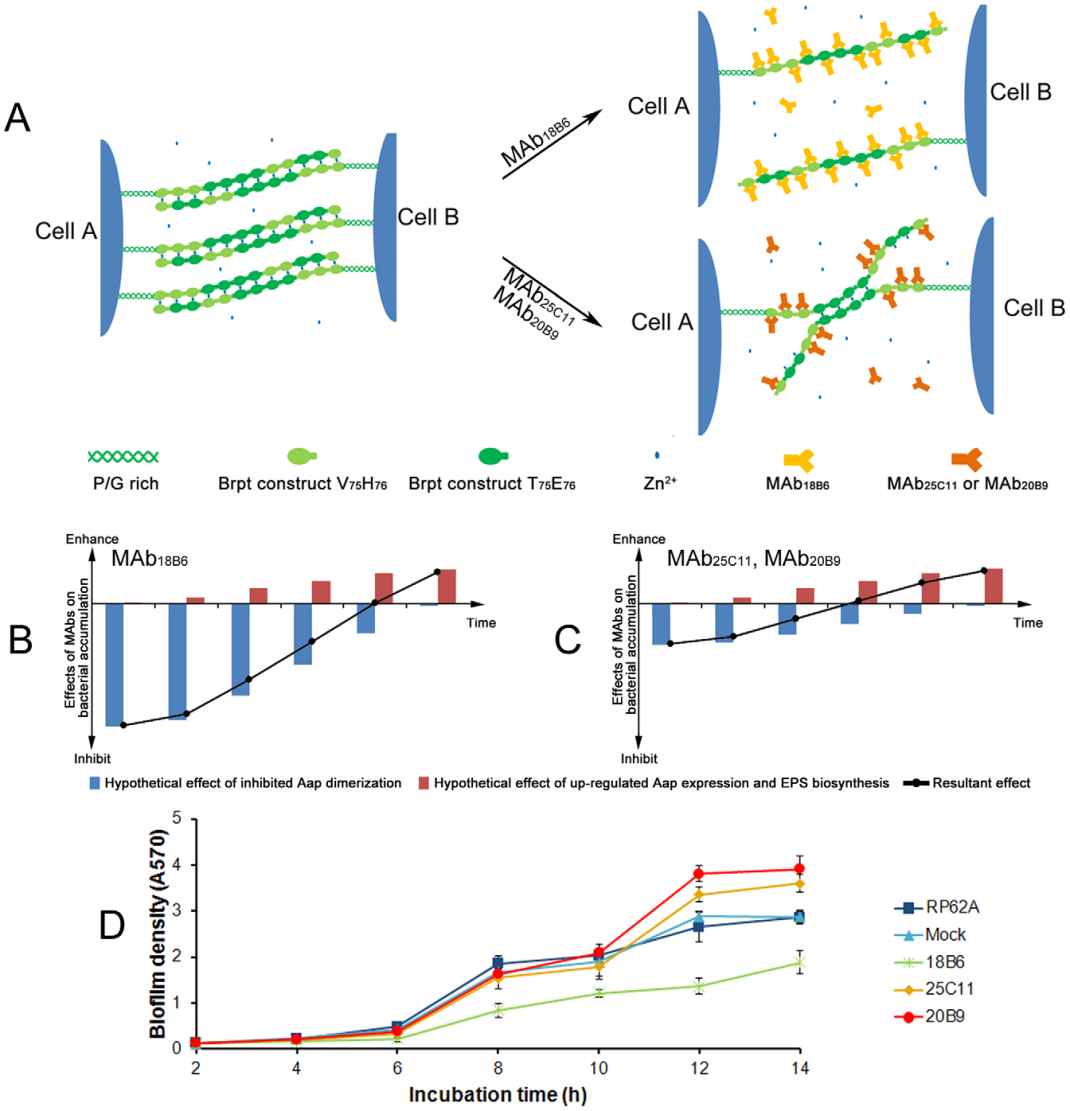
Contradictory actions of the MAbs on biofilm formation. (A) Model for MAbs affecting Aap dimerization. According to the zinc zipper model for intercellular adhesion mediated by zinc-dependent Aap dimerization [22], we speculate that MAb_18B6_ binds to all twelve AapBrpt constructs and significantly inhibits Aap dimerization, whereas MAb_25C11_ and MAb_20B9_ bind to six of the AapBrpt constructs and block Aap dimerization incompletely. (B, C) Model for MAbs affecting bacterial accumulation. The resultant effect (black curves) of the MAbs on bacterial accumulation is attributed to the counteraction between two contradictory actions, one inhibits biofilm formation by binding to Aap (blue bars), and the other enhances bacterial accumulation by up-regulating Aap expression and EPS biosynthesis (red bars). (D) Kinetic studies on biofilm formation. The cells of *S. epidermidis* RP62A were co-cultured with 10 µg/mL of each MAb in 96-well polystyrene plates initially at 4°C for 2 h, and then at 37°C for 2 h, 4 h, 6 h, 8 h, 10 h, 12 h, and 14 h, respectively. After incubation, biofilm formation at different time points was measured using crystal violet staining, and the results are depicted as means ± SD of three independent experiments.

Overall, the resultant effect of the MAbs on biofilm formation is attributed to the counteraction between these two actions. At the early phase of culture, MAb_18B6_ binds to all twelve AapBrpt constructs and then significantly inhibits Aap dimerization and bacterial accumulation (Figure 10A). With its inhibition of Aap dimerization overwhelming the action of up-regulated Aap expression and EPS biosynthesis (Figure 10B), MAb_18B6_ inhibits biofilm formation. However, MAb_25C11_ and MAb_20B9_ only bind to six of the AapBrpt constructs and block Aap dimerization incompletely (Figure 10A). Consequently, their weak inhibition of Aap dimerization fail to overcome the action of up-regulated Aap expression and EPS biosynthesis (Figure 10C), MAb_25C11_ and MAb_20B9_ show little inhibition on biofilm formation. When it comes to the late phase, the MAbs almost have been degraded completely, and the up-regulated Aap expression and EPS biosynthesis starts to play a leading role, which aggregates the planktonic to form cell clusters (at 9 h post-incubation) and enhances the biofilm formation (Figure 10B, C).

Therefore, at 14 h post-incubation, the bacteria co-cultured with MAb_18B6_ shows weaker biofilm production, resulting from slight reinforcement of the biofilm development which has been severely suppressed in early phase (Figure 10B). Meanwhile, due to the weak inhibition of Aap dimerization in early phase and the intense up-regulation of Aap expression and EPS biosynthesis (especially for eDNA release) in late phase, MAb_25C11_ and MAb_20B9_ eventually lead to an enhanced biofilm formation (Figure 10C). By dynamic monitoring the biofilm formation, we found that the kinetic biofilm formation of the bacteria co-culture with the MAbs is in agreement with our models described above. The inhibited biofilm development of the bacteria co-cultured with MAb_18B6_ was slightly reinforced after 12 h post-incubation, while the uninhibited biofilm formation of the bacteria co-cultured with MAb_25C11_ and MAb_20B9_ was strengthened after 10 h post-incubation (Figure 10D).

The enhancement of *S. epidermidis* biofilm formation by anti-Aap MAbs was also reported by Broekhuizen and *et al* [37]. They found that the MAbs may increase rather than reduce binding of *S. epidermidis* to biomaterials in an in vivo model. These antibodies may have similar characteristics to our MAbs, which enhance the biofilm formation by up-regulating Aap expression and EPS biosynthesis. Considering those findings, vaccine development using Aap as the antigen should pay attention to the following points: which is the optimum epitope that mediate the highest inhibition of Aap dimerization; how to reduce the effect of anti-Aap antibodies on up-regulating Aap expression and EPS biosynthesis.

In conclusion, in addition to our novel findings on the altered Aap expression and EPS biosynthesis in *S. epidermidis* mediated by mouse MAbs against Aap, the epitope mapping of biofilm-affecting MAbs will, for the first time, contribute to a better understanding of staphylococcal biofilm formation and help to develop epitope-peptide vaccines against staphylococcal infections.

## Materials and Methods

### Ethics statement

All procedures performed on mice were conducted according to relevant national and international guidelines (the Regulations for the Administration of Affairs Concerning Experimental Animals, China, and the NIH Guide for the Care and Use of Laboratory Animals) and were approved by the Institutional Animal Care and Use Committee (IACUC) of Shanghai Medical College, Fudan University (IACUC Animal Project Number: 20090613-qu).

### Materials

Prokaryotic vectors expressing AapBrpt1.5 and its truncated forms were pSJ8 (MBP-His fusion vector) and pET28a (+), respectively. The DNA fragment encoding GB1 tag [24], [25], [26] which fused at the N-terminal of the truncated AapBrpt1.5 was cloned from pETMG (GB1-His fusion vector). MAbs against AapBrpt1.5 were prepared by Abmart Inc. (Shanghai, China; http://www.ab-mart.com). *S. epidermidis* strains RP62A and ATCC 12228 were purchased from the American Type Culture Collection (ATCC, http://www.atcc.org), *S. epidermidis* strain 1457 was kindly provided by Dr. Yicun Gao from Hong Kong University, and clinical strains of *S. epidermidis* were isolated from patient samples from Zhongshan Hospital and Ruijin Hospital in Shanghai, China. All of the oligonucleotide primers used in this study are listed in Table 5.

**Table 5:** Oligonucleotide primers used in the present study.

Primers	Sequence (5′→3′)
**Primers used for PCR amplification of AapBrpt1.5^a^**	
F	CGCGGATCCCCAGTTGATGGAGATCCGATTAC
R	CCGCTCGAGTTATTTTGTTGGACCATACTCAACAATT
**Primers used for Q-PCRs in eDNA quantification^b^**	
*gyrA* F	CCTTATGAAACTCGGAGATGG
*gyrA* R	TCAGTAGTAGTAGATTGTTGCG
*serp0306* F	ATGCCACATCCACGAAAGA
*serp0306* R	TGTAACTGACAATGCCCAATC
*lysA* F	TGACAATGGGAGGTACAAGC
*lysA* R	TGGTCTTCATCGTAAACAATCG
*leuA* F	GTGAACGGTATTGGTGAAAGAG
*leuA* R	GTGGTCCTTCCTTACATATAAAGC
**Primers used for Q-RT-PCRs in Aap transcriptional analysis^b^**	
*gyrB* F	AGAAGAGGAAGTTAGAGAAGA
*gyrB* R	GCATATCCACTGTTATATTGAAG
*aap* F	ACGAGGAATTACAATCATCA
*aap* R	GTAGTTGGCGGTATATCTATT
**Primers used for truncation of the GB1-His-tagged AapBrpt1.5**	
F^c^	CATGCCATGGGCATGCAGTACAAGCTTGCTCTGAACG
*TF_1–160_* R^a^	CCGCTCGAGTTAAACGCGCTCTTCACCTGGTTTTAAA
*TF_1–102_* R^a^	CCGCTCGAGTTAAACTTCAGTTTTACTATCTACAGGTGCA
*TF_1–53_* R^a^	CCGCTCGAGTTATGTTAATGGGTTCTTAGTTGTTGGC
*TF_1–132_* R^a^	CCGCTCGAGTTAACCAACTTTCGGACCATATTTTGT
*TF_1–122_* R^a^	CCGCTCGAGTTAATCCACTGGTGGGGTAACAACT
*TF_1–112_* R^a^	CCGCTCGAGTTAATCAGGATTTTTAACTCCTGGTTTA
*TF_1–90_* R^a^	CCGCTCGAGTTAAAATTCATCTTTATGACCTTGTGGTAT
*TF_1–80_* R^a^	CCGCTCGAGTTATTCACCACCATAATGAACAATCTCAT
*TF_1–70_* R^a^	CCGCTCGAGTTATGGTTGTTTTGTTATTTTTTCTGTTGG
*TF_1–60_* R^a^	CCGCTCGAGTTAACCTTCGCCAACTTTTTCTCCTGTT
**Primers used for site-directed mutagenesis of the GB1-His tagged TF_1_**_*–***102**_	
F^a^	ACAACCAGTGGATGAGATTACTGAATATGGTGGTGAACAAATACC
R^a^	GGTATTTGTTCACCACCATATTCAGTAATCTCATCCACTGGTTGT

**a**. Primers were designed according to the genomic sequence of *S. epidermidis* ATCC 12228 (GenBank NC_004461).

**b**. Primers were designed according to the genomic sequence of *S. epidermidis* RP62A (GenBank NC_002976).

**c**. Primers were designed according to the gene sequence of the 56-residue B1 immunoglobulin binding domain (GB1) of immunoglobulin G-binding protein from *Streptococcus dysgalactiae* subsp. *equisimilis* GGS_124 (amino acids 303–357, GenBank YP_002997067).

### Expression and purification of recombinant AapBrpt1.5

The gene coding for AapBrpt1.5 (amino acids 1088–1296, GenBank NP_763730) was PCR-amplified from genome of *S. epidermidis* ATCC 12228. AapBrpt1.5 was expressed as a fusion protein with an N-terminal maltose-binding protein-tagged six-histidine (MBP-His) tag. The MBP-His-tagged AapBrpt1.5 was purified using a Ni-NTA column (Qiagen, http://www.qiagen.com) and then cleaved using TEV protease. The un-tagged AapBrpt1.5 was further purified using Ni-NTA and Superdex 75 gel filtration columns (GE Healthcare, http://www.gehealthcare.com). The molecular mass of the untagged AapBrpt1.5 was verified using the LC/ESI-MS system (Agilent, http://www.agilent.com, and Thermo Finnigan, http://www.thermoscientific.com).

### Expression of truncated AapBrpt1.5

To perform the epitope mapping for the anti-AapBrpt1.5 MAbs, AapBrpt1.5 was truncated using the following methods. Briefly, AapBrpt1.5 was first N-terminally fused with a GB1-tagged six-histidine (GB1-His) tag [24], [25], [26], and the DNA fragment coding for truncated AapBrpt1.5 with the N-terminal GB1-His tag was then amplified using PCR and further cloned into vector pET28a(+) to be used for expression of the truncated fragments.

### Immunoprecipitation

The purified proteins were diluted in lysis buffer containing 20 mM Tris-HCl (pH 8.0), 137 mM NaCl, 1 mM EDTA, 1% (vol/vol) Triton X-100, and protease inhibitors for non-denaturing buffer conditions or 1% (wt/vol) SDS, 5 mM EDTA, 10 mM beta-mercaptoethanol, and protease inhibitors for denaturing buffer conditions. The samples for the denaturing immunoprecipitation were further diluted with the non-denaturing lysis buffer. The immunoprecipitation was performed using the MAbs and protein G-coupled Sepharose beads (Santa Cruz, http://www.scbt.com). Normal mouse IgG (Santa Cruz, http://www.scbt.com) was used as the negative control. After the immunoprecipitation, the beads were washed with the non-denaturing lysis buffer and boiled in SDS sample buffer, and a rabbit anti-His-tag antibody (Cell Signaling Technology, http://www.cellsignal.com) was used to detect the immunoprecipitated proteins.

### Western blot

Bacterial lysates and immunoprecipitated samples were prepared in SDS sample buffer, and the proteins contained in the samples were separated using SDS-PAGE (7% or 12%). Separated proteins were further electrotransferred onto a polyvinylidene fluoride (PVDF) membrane (Millipore, http://www.millipore.com) and probed with the corresponding primary antibodies: anti-AapBrpt1.5 MAbs (1 ng/mL) or rabbit anti-His-tag antibody. After incubation with the HRP-conjugated secondary antibody (Santa Cruz, http://www.scbt.com), the immunoreactivity of the membranes was visualized using an ECL Western blotting system (Thermo, http://www.thermo.com).

### Detection of biofilm formation

Biofilm formation was detected using a semiquantitative plate assay [38]. An overnight culture of *S. epidermidis* grown in TSB was diluted 1∶200 into fresh TSB containing dilutions of mouse serum or MAbs, and statically incubated in 96-well polystyrene plates (Corning, http://www.corning.com), initially at 4°C for 2 h and then at 37°C for 14 h. After incubation, the wells were washed with PBS, fixed with methanol, and stained with 2% (wt/vol) crystal violet. The absorbance of the wells was determined at 570 nm using a 96-well plate spectrophotometer (Beckman Coulter DTX880, http://www.beckmancoulter.com). For microscopic observation of biofilm formation, *S. epidermidis* biofilms were further cultivated in FluoroDishes (FD35-100, WPI, http://www.wpiinc.com) [28], stained with a Live/Dead kit (containing SYTO9 and PI, Invitrogen, http://www.invitrogen.com), and observed under a confocal laser scanning microscopy (TCS SP5 CLSM, Leica, http://www.leica-microsystems.com).

Disintegrating treatment of the biofilms and the bacterial cell clusters were tested as published elsewhere [17], [27], [39], [40]. The biofilms were formed in the presence of 0.14 U/µL DNase I (Takara, http://www.takara-bio.com) for 14 h, and then washed with PBS, fixed with methanol, and stained with 2% (wt/vol) crystal violet. For disintegrating treatment of the bacterial clusters, the clusters were treated with 0.1 mg/mL proteinase K (Merck, http://www.merck.com), 20 mM Tris (pH 7.5), 100 mM NaCl for 30 min at 37°C, 10 mM sodium metaperiodate, 50 mM sodium acetate (pH 4.5) for 6 h at 37°C, or 0.2 U/µL DNase I in PBS for 6 h at 37°C.

### RNA extraction and quantitative RT-PCR

After static incubation at 37°C for 14 h in 50-mm polystyrene dishes, *S. epidermidis* cells (planktonic and biofilm) were collected and washed with ice-cold saline, and then homogenized using 0.1 mm Ziconia-silica beads in Mini-Beadbeater (Biospec, http://www.biospec.com) at a speed of 4800 rpm. The bacterial RNA was isolated using an RNeasy kit (QIAGEN, http://www.qiagen.com) according to its standard protocol. The relative expression level of the *aap* gene was determined by quantitative RT-PCR with gene-specific primers listed in table 5.

### Immunofluorescence assay

Biofilms of *S. epidermidis* RP62A were cultured on glass coverslips. After static co-incubation with or without the MAbs at 4°C for 2 h initially and then at 37°C for 14 h, the coverslips with the biofilms were washed gently with PBS, fixed with methanol, and blocked with 3% (wt/vol) bovine serum albumin (BSA) in PBS for 4 h at room temperature. Antigens contained in the biofilms were primarily probed with 10 ng/mL MAb_25C11_ or 1∶400 diluted mouse anti-*S. epidermidis* serum and secondarily probed with Cy3-conjugated goat anti-mouse secondary antibody (Jackson ImmunoResearch, http://www.jacksonimmuno.com). After three washes with PBS, the biofilms were further stained with SYTO9 (1 µM, Invitrogen, http://www.invitrogen.com). The antigen distribution was observed under a Leica TCS SP5 CLSM.

### PIA quantification

PIA was quantified as previously described [30], [31], [32]. In brief, after static incubation at 37°C for 14 h in 35-mm polystyrene dishes, *S. epidermidis* cells were scraped off and resuspended in 0.5 M EDTA (pH 8.0). After treatment with proteinase K (4 mg/ml; Merck, http://www.merck.com) for 3 hours at 37°C, serial dilutions of the PIA extracts were spotted onto nitrocellulose transfer membranes (Millipore, http://www.millipore.com). The air-dried membrane was blocked with 3% (wt/vol) BSA and subsequently incubated with 3.2 µg/mL horseradish peroxidase-coupled wheat germ agglutinin (WGA-HRP conjugate; Lectinotest Laboratory, National Medical University in Lviv, Ukraine) for 1 h. HRP activity was visualized using chromogenic detection.

### Extracellular DNA quantification

The isolation of eDNA from biofilms was performed as described previously [27], [28], [29]. After static incubation at 37°C for 14 hours, the 96-well polystyrene plates were chilled at 4°C, and EDTA was added to each well to a final concentration of 2.5 mM. The supernatants were discarded, and the unwashed biofilms were harvested by resuspension in 50 mM Tris-HCl (pH 8.0), 10 mM ETDA, and 500 mM NaCl. After centrifugation, the supernatant was diluted in 10 mM Tris-HCl (pH 8.0), 1.0 mM EDTA (TE) and extracted with phenol/chloroform/isoamyl alcohol (25∶24∶1) and chloroform/isoamyl alcohol (24∶1). DNA in the aqueous phase of each sample was then extracted using ethanol precipitation and dissolved in TE buffer. Quantitative PCRs were performed using SYBR Premix Ex Taq (Takara, http://www.takara-bio.com) on 1∶10 dilutions of each sample using the four primer sets listed in Table 5.

### Triton X-100-induced autolysis

Autolysis of *S. epidermidis* RP62A treated with the MAbs was analyzed according to previously published methods [41]. An overnight culture of *S. epidermidis* RP62A was diluted 1∶200 into fresh TSB supplemented with 0.1 mM NaCl and 10 µg/mL MAbs and incubated at 4°C for 2 h then shaken at 37°C until the A_600_ reached 0.8. The collected bacteria were washed with distilled water and resuspended in 50 mM Tris-HCl (pH 7.2), and 0.05% (vol/vol) Triton X-100. The resuspended bacteria were shaken at 30°C for an additional 2 h, and autolysis was monitored by measuring the turbidity of the suspension at A_600_ every 15 min.

### Site-directed mutagenesis

Site-directed mutagenesis was performed with the QuikChange site-directed mutagenesis kit (Stratagene, Agilent Technologies, http://www.genomics.agilent.com), using the plasmid used for expression of the truncated AapBrpt1.5 fragment TF_1*–*102_ as a template. The primers used for the mutant (V_75_H_76_ to T_75_E_76_) are listed in Table 5. The mutant plasmid was confirmed by DNA sequencing before being transformed into *E. coli* strain BL21(DE3).
